# Pain Neuroscience Education: State of the Art and Application in Pediatrics

**DOI:** 10.3390/children3040043

**Published:** 2016-12-21

**Authors:** Hannah Robins, Victoria Perron, Lauren C. Heathcote, Laura E. Simons

**Affiliations:** 1 Division of Pain Medicine, Department of Anesthesiology, Perioperative and Pain Medicine, Boston Children’s Hospital, MA 02115, USA; hrobins4@binghamton.edu (H.R.); perronv@bc.edu (V.P.); 2 Department of Anesthesiology, Perioperative and Pain Medicine, Stanford University School of Medicine, 1070 Arastradero Road, Palo Alto, CA 94304, USA; lcheath@stanford.edu

**Keywords:** pain neuroscience education, psychoeducation, cognitive intervention, biopsychosocial model, pediatric chronic pain

## Abstract

Chronic pain is a widespread problem in the field of pediatrics. Many interventions to ameliorate pain-related dysfunction have a biobehavioral focus. As treatments for chronic pain (e.g., increased movement) often stand in stark contrast to treatments for an acute injury (e.g., rest), providing a solid rationale for treatment is necessary to gain patient and parent buy-in. Most pain treatment interventions incorporate psychoeducation, or pain neuroscience education (PNE), as an essential component, and in some cases, as a stand-alone approach. The current topical review focuses on the state of pain neuroscience education and its application to pediatric chronic pain. As very little research has examined pain neuroscience education in pediatrics, we aim to describe this emerging area and catalyze further work on this important topic. As the present literature has generally focused on adults with chronic pain, pain neuroscience education merits further attention in the realm of pediatric pain in order to be tailored and implemented in this population.

## 1. Introduction

Pediatric chronic pain has reached epidemic proportions with an estimated 1.7 million children in the USA alone suffering from moderate to severe persistent pain [1]. With the high number of children affected, it is of extreme importance to discover new, innovative methods of treatment for the pediatric population. One approach that has been researched and implemented in adult populations over the past several decades is psychoeducation, either as one element of a comprehensive treatment program or as a stand-alone intervention. By explaining the scientific concepts that are central to the pathogenesis and perpetuation of chronic pain, clinical providers hope to create lasting change in the patient’s beliefs about pain, and in turn, increase their engagement in the biobehavioral recommendations made for pain management and reduction. Pain education addresses patient misconceptions about physiological phenomena and helps shift their perspective to the idea that pain is dependent on biological, psychological, and social processes. One common message is that pain is dependent on meaning [2], and that how the patient perceives their pain is key to how a patient’s brain processes pain signaling [3]. Taken together, pain education programs center on an explanatory model that understanding pain can modify pain itself.

Several terms have been coined in relation to pain education with each aiming to convey the central idea of a given approach. They include psychoeducation, pain neuroscience education (PNE), pain biology education, therapeutic neuroscience education, and Explain Pain (EP). For simplicity, we will refer to these approaches broadly as pain neuroscience education (PNE) [4], unless describing a study where a specific approach was empirically tested. PNE can be taught as an intervention on its own, as well as in combination with another form of therapy (such as cognitive-behavioral or physical) [5]. Although it is likely that all patients suffering with chronic pain can benefit from a shift in mindset provided via PNE, it might be critical for patients who suffer from a centralized pain problem and/or struggle with maladaptive perceptions about their pain [6]. This paper reviews the current state of the art in pediatric PNE. As most PNE research has been conducted in adult populations to date, we discuss both adult and pediatric studies within each section, considering how adult studies can inform future pediatric research, and presenting next steps for the pediatric pain field.

## 2. Why Pain Neuroscience Education?

### 2.1. Contextual Information about Pain Influences Pain Perception

When examining the concept of PNE, it is helpful to look to experimental research to determine the theoretical efficacy of education in altering pain outcomes. When we educate patients about pain, this new information alters the context in which they perceive their pain. In adult non-patient populations, research has demonstrated that manipulation of information and context regarding a stimulus can modulate pain. In one adult study using a cold pressor test (CPT), stimulus information was manipulated by having one group read a threatening passage about frostbite, while another read a passage about the safety of the test and how pain can be unrelated to tissue damage [7]. They found that the group that read the threatening passage had a lower mean pain tolerance time. In another adult study, investigators examined the idea that heat can be perceived to be more tissue-damaging than cold [8]. A −25 °C metal stick was placed on participants’ necks after telling them that it was either hot or cold. Those who were told that the metal bar was hot rated both the painfulness and the damaging properties of the stimulus higher than participants who were told the metal bar was cold. In a third adult study, it was shown that using the colors red and blue as visual cues before presentation of a stimulus modulated pain intensity [9]. A red visual cue indicating heat resulted in higher pain ratings than a blue visual cue indicating cold.

In pediatrics, the manipulation of information and context has also been shown to modulate pain expectations and emotional response to pain. In one study [10], non-patient children completing a CPT received either threatening CPT information (CPT described as very painful, high pain expressions depicted) or non-threatening CPT information (standard CPT instructions provided, low pain expressions depicted). Children in the high-threat condition expected more pain, perceived the pain as more threatening, and catastrophized more about the pain. Parents of children in the high threat condition also expected their child to experience more pain. In another, parent-focused study [11], parents received either threatening information (stimulus described as painful and barely tolerable, high pain expressions depicted) or non-threatening information (stimulus described as slightly unpleasant, low pain expressions depicted) about a heat stimulus their child would receive. Parents who received the threatening information showed stronger negative physiological responses (EMG corrugator activity and fear-potentiated startle reflex) to cues of upcoming child pain. This was particularly the case when the child’s pain facial expression was high, suggesting that parent beliefs are impacted by information from the environment and from the child.

With regards to patient populations, many individuals suffering from chronic pain can develop irrational beliefs and fears (including catastrophizing) about their pain. Many patients believe that their pain is harmful to their bodies and associate it with danger, despite the absence of tissue damage. These associations, similar to a red light visual cue or a passage about the dangers of frostbite, may worsen their pain. In adult patient populations, it has been shown that exposing patients to inaccurate information regarding illness may harm health outcomes and care [12]. Moreover, adult patients who are unsure of the diagnosis of their pain problem or perceive their pain as enduring and mysterious display higher levels of catastrophizing and are less likely to use coping strategies to deal with their pain [13,14].

In pediatrics, qualitative research also indicates a negative impact of diagnostic uncertainty and inaccurate information provision on child and parent pain and medical experiences. Children who are better informed about a forthcoming medical procedure are generally shown to have better outcomes (lower distress and better adjustment) during and after the procedure [15]. For parents, feeling uncertain with regards to their child’s chronic pain condition and prognosis is shown to relate to parental feelings of helplessness and distress [16]. Studies are now needed to directly examine the effects of information provision in pediatric clinical populations. Due to the important role of child and parent beliefs and fears, it is crucial that healthcare professionals assess and appreciate these beliefs in order to determine if PNE would be an appropriate and worthwhile intervention within the context of pediatric pain. 

### 2.2. Pain Neuroscience Education Provides a Common Language between Provider and Patient

A gap in understanding and communication between the patient and doctor can be a barrier in the treatment of chronic pain. PNE may provide a common language to aid communication and understanding. However, doctors’ expectations of patient understanding and actual patient ability to understand must align for patients to receive helpful information about pain. A study with adults used the Neurophysiology of Pain Test to assess the ability of patients and healthcare professionals to accurately understand the neurophysiology of pain, as well as healthcare professionals’ perceptions of the ability of patients to understand the concepts [17]. The results showed that after an education session, both groups were able to understand the information, but healthcare professionals significantly underestimated the ability of patients to do well on the Neurophysiology of Pain Test.

In pediatrics, discrepancies between doctors’ expectations of patient understanding and actual patient ability to understand may be particularly pertinent, as patients’ cognitive capacity changes across development, and does not always align with child age. The effect of age and cognitive capacity on PNE efficacy is currently unknown. However, a 2007 review acknowledged the importance of cognitive-developmental considerations within the provision of information regarding pediatric medical procedures [15]. Research has also indicated communication issues between child chronic pain patients and doctors, as well as discrepancies between doctor information provision and patient needs. The oral testimonies of patients at the Pediatric Pain Clinic at UCLA revealed patient frustration regarding the doctor’s lack of interest in their experience [18]. The testimonies suggested a fundamental difference in the language and orientation of patients and doctors in regards to pain. While the child’s orientation was experiential and emotional, the doctor’s was instructive and diagnostic. This suggests that changes are needed in the communication models employed in treating pediatric chronic pain so that information being exchanged between treatment providers and patients begins to resonate. In addition, if providers assume their patients will not understand pain neuroscience information, they will not attempt to include it in appointments or treatment sessions. Thus, it is necessary for providers to be well-versed in PNE and to feel comfortable delivering that information in a developmentally appropriate way. 

## 3. Overview of Pain Neuroscience Education

### 3.1. Explanatory Models

There are several theoretical models that have emerged to explain chronic pain, many of which have already been applied to a pediatric population. These models outline the concepts that are the foundation of PNE, provide physicians with a way to conceptualize different aspects of the patient’s pain (including the obstacles that patients must overcome), and offer scaffolding for researchers formulating new research questions. As more information is learned over time about pain, the components of these theoretical models have evolved. Here we describe each model in turn, before presenting their pediatric application, where evident.

Originally, pain was viewed within a biomedical model. This model assumes a one-to-one correspondence between tissue damage, nociceptive input, and pain sensation. Thus, pain was thought to be a direct response to injury, and psychological or behavioural issues were thought to arise as a consequence of pain but not to influence the pain itself [19]. However, by the late 20th century, scientists began to shift away from this idea and moved towards the concept that motivational, affective, and cognitive processes can modulate pain, and can in some cases be the initial factor in pain etiology [20]. This is especially relevant to those suffering from chronic pain, as the pain often emerges despite seemingly normal biological functioning. The biomedical model has now largely been replaced with a biopsychosocial model, which incorporates all of the aspects in a patient’s life that converge and potentially maintain a cycle of pain [21]. This has been determined to be a more effective conceptual and theoretical stance as it is suggested that pain cognitions are not only associated pain intensity, but may also be barriers to effective treatment if left unaddressed [22].

The biopsychosocial model puts an emphasis on the fact that ‘pain itself is modulated by beliefs… and can therefore be improved by modifying inaccurate beliefs’ [2]. In order for this model to be successful, health-care providers need to convey the multi-dimensional causes of pain that are to be tackled in the intervention. The biological component of the biopsychosocial model largely revolves around the complex interplay of several cortical and subcortical brain regions involved in sensory, motor, cognitive, affective, and motivational functions [23]; with recent data suggestive of global brain connectivity reorganization among chronic pain patients [24]. This bombardment of neural input is a key mechanism that leads to chronic pain, as the brain keeps sending pain signals even in the absence of tissue damage. One theory that centers on the psychological aspect of the biopsychosocial model, entitled The “Common Sense Model of Self-Regulation” [25] highlights health beliefs and builds a hierarchical framework of patient cognition [26]. The model details the individual’s representation of a health threat and the factors that contribute to this representation. It describes the way in which a person responds to any given health threat, including cognitive and emotional processes. Five dimensions of these cognitions have been identified and include: (1) identity (the effort to evaluate symptoms and label the illness); (2) cause (the subjectively formulated belief of what is causing the symptoms); (3) time-line (the patient’s perception of how long the problem will last); (4) consequences (the patient’s predictions of how the illness will affect them in different areas of their life); and (5) controllability (the patient’s belief regarding their outcome and personal ability to change it) [2,3,27]. PNE, being closely tied to cognitive-behavioral treatments, shares significant ideology with the Common Sense Model of Self-Regulation. PNE aims to help patients reevaluate their pain problem, to target beliefs in order to develop more effective coping skills, and to ultimately change each of the five cognitive dimensions to achieve a positive outcome. The Common Sense Model has been said to be instrumental in the foundation of many cognitive treatments [26], and has been used in a randomized controlled trial of pain education in adults with cancer [28].

**Figure 1:**
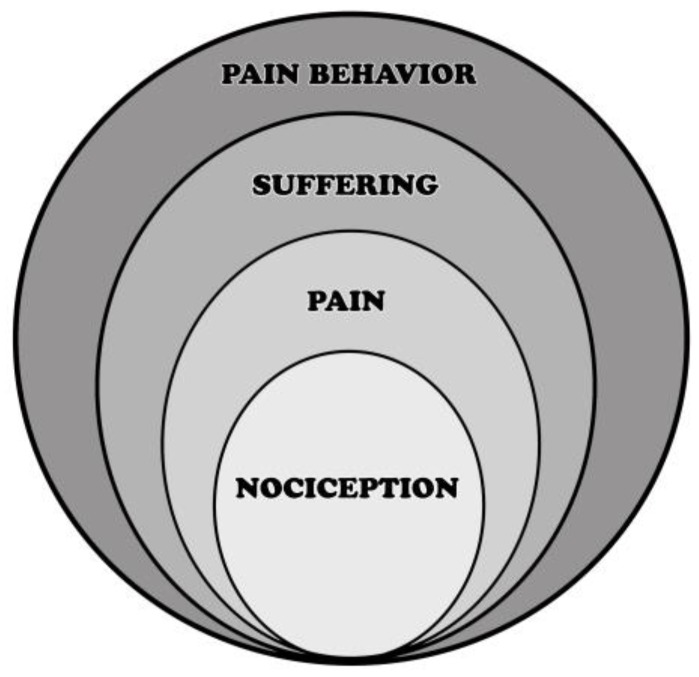
Loeser’s onion model displaying the four components of pain phenomena. Reprinted from Loeser, J.D. Pain as a Disease [29].

In addition to the comprehensive biopsychosocial model, additional theoretical models have emerged that highlight and attempt to explain in greater detail the different aspects of this larger framework. John D. Loeser has described pain as an onion consisting of four layered components: nociception, pain, suffering, and pain behavior [29] (Figure 1). The lower layers of suffering, pain, and nociception are not visible on the outside, being private experiences that only the patient is subjected to. The exterior of the onion is pain behavior, which is how the individual expresses his/her pain to the public. This could be through words, actions, or expressions. The onion model illustrates that in order to deliver effective treatment, the patient’s hidden layers must be acknowledged and understood.

Among several cognitive-affective processes at work in the context of chronic pain, none have received greater research [30] and clinical attention [31] than pain-related fear. This is likely due to the inherently adaptive nature of fear in response to a noxious stimulus. The Fear-Avoidance Model (FAM) [30,32] details the cycle of pain-related fear and activity avoidance that ultimately leads to functional disability. As we adopt a biopsychosocial stance on the persistance of chronic pain, fear is argued to influence patient motivations, decisions, and well-being. For some individuals, breaking a vicious cycle of fear and avoidance will necessitate an extensive and thorough PNE. Through learning new information about the biology of pain, patients may be able to rework their relationship to their pain and change their maladaptive and fearful response to an adaptive and flexible one, eventually leading to a better quality of life [33].

#### Application of Explanatory Models to Pediatrics

**Figure 2:**
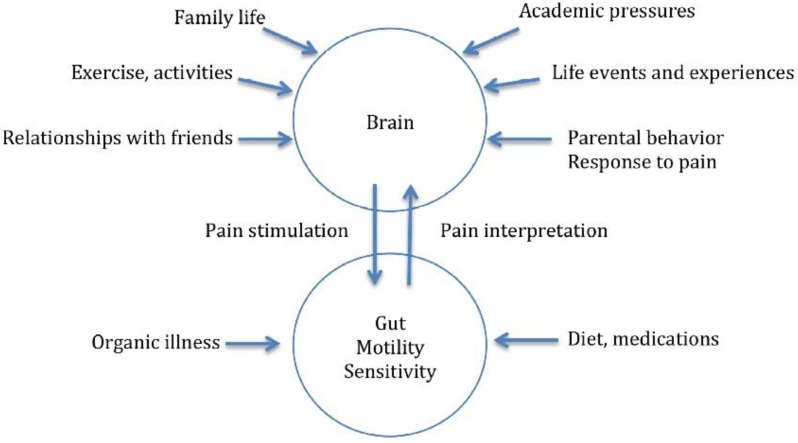
Visual representation of the biopsychosocial model in the context of pediatric functional abdominal pain. Adapted from Brown, L.K.; Beattie, R.M.; Tighe, M.P. Practical management of functional abdominal pain in children [35].

The models described above are often used in pediatric clinical settings [34], although there is little published research presenting adaptations of these models for pediatric delivery. The importance of the biopsychosocial model was highlighted in a recent case study of a nine-year-old girl with functional abdominal pain [35]. In this case, it was explained to the patient that pain does not necessarily require a noxious stimulus and can be modulated by experience and context. By using diagrams and age-appropriate metaphors (Figure 2), the complex scientific topics were put into simpler terms and provided the patient and her family concrete reasoning for her persistent pain state. 

**Figure 3:**
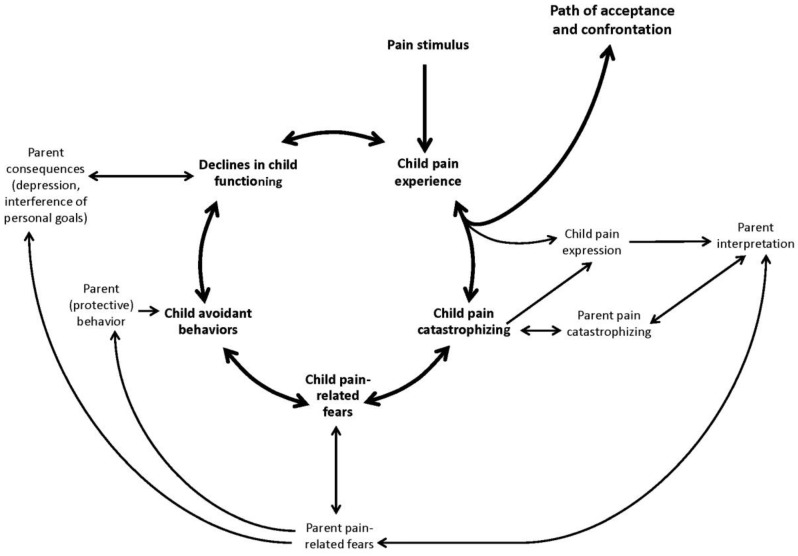
The interpersonal fear-avoidance model of chronic pain. Reprinted from Simons, L.; Smith, A.; Kaczynski, K.; Basch, M. Living in fear of your child’s pain: The parent fear of pain questionnaire [38].

The Common Sense Model has also been explored in youth with type 1 diabetes and sickle cell disease, conditions which are often accompanied by frequent pain [36]. However, it has yet to be well applied to other pediatric chronic pain populations. Perhaps the most well-adapted model for pediatric populations is the Fear Avoidance Model (FAM), which has been adapted [37] and validated [38] for youth with chronic pain (Figure 3). In particular, the pediatric FAM considers the important influence of parents for the child pain experience, including parental cognitions, affective responding, and coping behaviors. In a first empirical study, child FA factors were shown to be a good predictor of functional disability in youth with chronic pain [39]. Interestingly, duration of pain contributed to the model for younger children, whereas pain-related fears were more influential for adolescent patients, highlighting the importance of developmental factors in the application of explanatory models to pediatric populations. 

### 3.2. Current Evidence for PNE among Adults

There have been several systematic reviews conducted for adult PNE research that point to potential areas for growth in the field. For example, in 2011 Clarke and colleagues [40] reviewed studies of PNE specifically for chronic low back pain, for which they included only two randomized controlled trials (RCTs). The review revealed very low quality evidence that PNE is beneficial for pain, physical functioning, psychological functioning, and social functioning in this population, although the authors acknowledge that the review was limited by the small number of studies. More recently, Louw and colleagues [41] conducted an additional systematic review with a broader scope. Specifically, they included 13 RCTs that examined the influence of PNE on chronic musculoskeletal pain conditions. Five trials demonstrated positive effects in decreasing pain ratings, while three trials showed no effectiveness. Interestingly, of the three studies that were identified to increase pain knowledge, two of them showed an increase in pain knowledge, as well as a positive effect on pain catastrophization. However, an increase in pain knowledge is yet to be shown to correlate with decreased pain and disability [41].

Overall, the evidence for the effectiveness of PNE in adult patient populations is modest, particularly for long-term outcomes. However, the field is rapidly gaining momentum with additional studies being done each year. Moreover, it may be that the impact of PNE is better measured via its influence on mediators (e.g., pain catastrophizing, fear of pain) that ultimately influence outcomes. Perhaps PNE does not demonstrate a direct impact on pain-related function, but rather exerts its influence via these key mechanisms of change that have a demonstrated impact on outcomes in the literature. 

#### Current Evidence for Pain Neuroscience Education in Pediatrics

To date, there are few studies that examine the utility of PNE in pediatric populations. There is preliminary evidence that psychoeducation may be efficacious for improving pediatric outcomes, however, most studies have investigated educational programs focused on pain management rather than explaining the biology and neuroscience of pain. In 2007, Abram and colleagues [42] randomized pediatric headache patients to receive either a traditional neurological examination only, or the examination alongside a group educational session. The education session comprised information regarding stressors contributing to pain, pharmaceutical and behavioral treatments, and guided relaxation skills practice. Patients receiving the additional educational session demonstrated greater gains in headache knowledge and required slightly less physician face-to-face time. Both groups experienced a sustained decline in headache-related disability. Whilst this study provides preliminary evidence that psychoeducation can increase knowledge about pain management, the study did not examine PNE specifically. 

Perhaps unsurprisingly, many Cognitive-Behavioral Therapy (CBT) programs for pediatric chronic pain are delivered within a psychoeducational frame. Interestingly, a small number of RCTs for psychological therapies in pediatric chronic pain have used psychoeducation as a control intervention (see [43]). These studies have typically revealed superiority of psychological therapies (CBT and internet-based self-help training) over education for primary outcomes of functional disability and pain symptoms. However, where information regarding education content was provided [44,45], it is clear that education again focused on pain management rather than PNE specifically. Studies of primary PNE interventions for pediatric populations, particularly with randomized controlled designs, are greatly needed to advance understanding of the efficacy of PNE for pediatric populations.

### 3.3. New Applications of PNE: Preoperative Preparation and Cancer Pain Treatment

**Figure 4:**
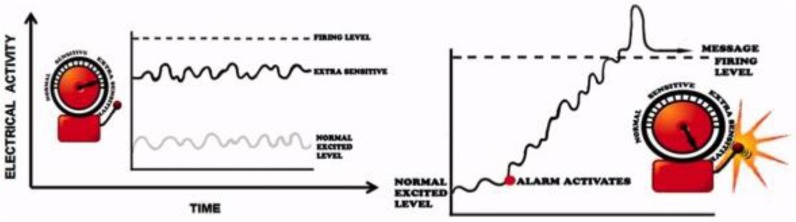
Example of illustration used in the PNET program to explain nervous system processes related to persistent pain. Reprinted from Louw, A.; Puentedura, E.J.; Diener, I.; Peoples, R.R. Preoperative therapeutic neuroscience education for lumbar radiculopathy: A single-case fMRI report [47].

Adult PNE research has rapidly expanded over the past decade, and has branched into new areas such as preoperative preparation and cancer treatment. For example, the Preoperative Neuroscience Education Tool (PNET) targets adult patients undergoing lumbar radiculopathy [46]. The goal of PNET is to reduce post-operative pain levels, catastrophizing, and disability, as well as increase physical performance. Since postoperative rehabilitation is often ineffective in reducing pain levels, preoperative education that addresses pain physiology by using illustrations (Figure 4), metaphors, and explanatory examples has been of recent interest.

Similar to PNET is the program RIDcancerPain (The Representational Intervention to Decrease Cancer Pain), that has been used as part of an RCT among adults with cancer pain [28]. RIDcancerPain aims to address the patient’s current beliefs about pain before changing their perceptions through a one-time educational program that introduces new concepts surrounding pain physiology and coping strategies. Results showed that patients receiving RIDcancerPAIN reported greater decreases in barriers to pain control and greater decreases in pain severity than those in the control group. This study is similar to a previous program on cancer pain management, which showed a moderately positive effect on pain intensity after patients watched an educational video and had an informal discussion with a nurse [48]. Although some elements diverge between palliative care and chronic pain management, the emphasis on doctor interaction and education on pain physiology highlighted in these studies demonstrates where PNE converges across patient populations.

In pediatrics, Chambers and colleagues [49] conducted a randomized trial of a pain education booklet for parents of children undergoing surgery. Parents receiving the pain education booklet, in comparison to those receiving a pain assessment control or no pain education, had fewer concerns about the use of analgesics for children, but there were no group differences in parents’ pain symptom assessments on any of the three days following surgery. Again, the pain education booklet focused on pain management strategies rather than explaining the neuroscience of pain. More recently, Tabrizi and colleagues [50] investigated the use of an anesthesia education booklet to alleviate preoperative anxiety in children ages 8–10 and their parents. Whilst parents and children receiving the education intervention reported reduced preoperative anxiety, similar reductions were seen in a control group receiving routine preoperative preparation without education. 

### 3.4. A Combined Approach of Pain Neuroscience Education and Physiotherapy

It has been suggested that PNE sessions alone may not be sufficient to reach efficacious outcomes. Multiple studies have thus examined the success of combining PNE with physical therapy and exercise [51]. In a recent adult study [52], patients with chronic low back pain (CLBP) (*n* = 30) underwent two sessions of PNE followed by 12 sessions of aquatic exercise, all in a small group setting. The combination of both approaches resulted in statistically and clinically significant reductions in pain and functional disability when compared to the control group (*n* = 32) that received only the aquatic exercise sessions. This extends the results of a similar study in adult patients with fibromyalgia (*n* = 58) where the combination of pool exercise and PNE was found to be more effective than physiotherapy alone [53].

This combined approach is not limited to aquatic exercises. Land-based physiotherapy programs have also been successful. This integrated approach has lowered pain and disability in a recent RCT among adults with CLBP when compared to a control group [54]. While the control group (*n* = 28) only received advice from their general practitioners, the experimental group (*n* = 29) had a four-week PNE course that incorporated trunk muscle training and a standardized home-exercise program. Another study (*n* = 41) was completed shortly after, and investigated the use of group or individual education when used in combination with motor control training [55]. The individual education group showed larger decreases in pain and disability than the control group. A similar program was implemented in Brazil, where patients (*n* = 79) received one hour of stretching alongside a physiotherapist and one hour of CBT-focused psychoeducation with a nurse and psychologist, weekly for eight weeks [56]. Over the eight weeks, patients’ pain intensity and disability levels significantly decreased. Although the latter study did not contain a control group and should thus be evaluated with caution, these studies highlight that PNE within the realm of a multidisciplinary cognitive-behavioral pain management program may enhance patient treatment in the future.

Not all trials have indicated better results when combining PNE with other treatments compared with PNE alone. For example, one recent study among adult patients with LBP found greater decreases in pain and increases in pain self-efficacy in patients who only received pain biology education (*n* = 18), compared to those who also received group exercise classes (*n* = 20) [51]. Although this was an unanticipated finding, the authors suggested that an exercise-only group would be important to tease apart findings in a future study. 

Again, pediatric research on the combined effects of PNE and physiotherapy is lacking. However, given promising findings from adult studies, and growing support for the effectiveness of physiotherapy within an interdisciplinary program for treating children with chronic pain [57], research in this area is warranted.

## 4. Delivery Methods

The way that pain education is delivered and presented to patients may be as important as the content itself. Evaluating patients’ individual needs and capacities for understanding PNE is important for PNE success. This may be especially pertinent in a pediatric setting, where PNE must be adapted to match patients’ cognitive capacities. There are many modalities already in use in both adult and pediatric clinical populations, giving healthcare professionals options in how to engage their patients, and giving patients resources they can utilize outside of a doctor’s office. Below we examine common PNE delivery methods, particularly considering their use in pediatric populations. 

### 4.1. Metaphor

When educating patients about pain, creative ways of explaining biological processes are necessary. This is especially relevant to the pediatric population, where traditional lectures or scientific models may be ineffective. Metaphor or story-telling as a way of discussing pain phenomena can be a helpful tool in PNE. A 2013 randomized controlled trial [58] found that adult chronic pain patients given a book of metaphors and stories to explain pain biology, Painful Yarns [59], had a larger increase in knowledge about pain biology and a larger decrease in pain catastrophizing compared to patients who were given a book about pain management. Interestingly, patients in the metaphor group reported reading an average of 82% of their book, as opposed to 47% for the control group, suggesting that metaphor not only has the potential to alter perceptions, but it is also more engaging than more traditional methods of delivery [58]. 

**Figure 5:**
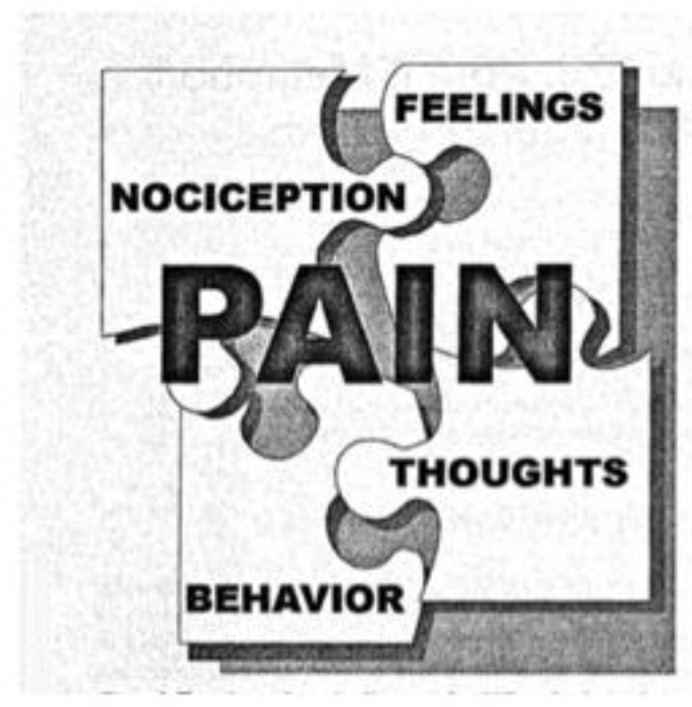
The pain puzzle visual metaphor. Reprinted from Rapoff, M.A.; Lindsley, C.B. The pain puzzle: A visual and conceptual metaphor for understanding and treating pain in pediatric rheumatic disease [59].

One metaphor proposed as a way of conceptualizing the pain problem for both patients and healthcare professionals is the pain puzzle (Figure 5); a visual and conceptual metaphor that identifies the multitude of factors that play into pain (nociception, affect/feelings, cognition/thoughts, and behavior). It can be explained that different individuals may have different pieces of varying sizes that make up their ‘personal pain puzzle’. The pain puzzle has been utilized in pediatric clinical settings for patients with rheumatic disease [60].

**Figure 6:**
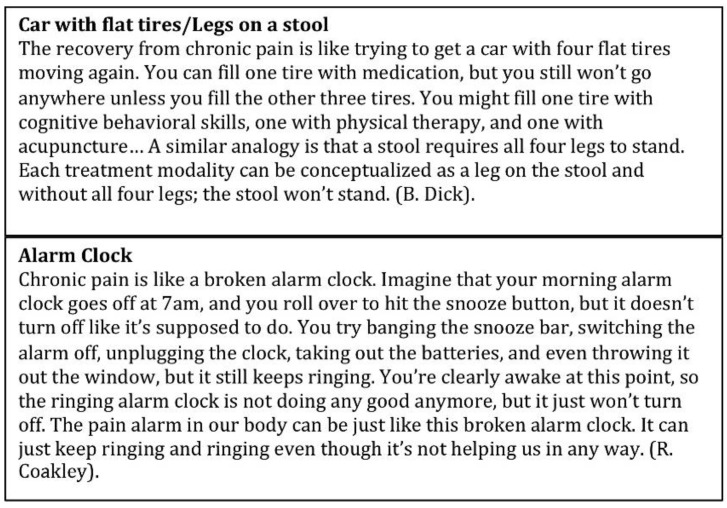
Example metaphors for explaining chronic pain to children. Adapted from Coakley, R.; Schechter, N.L. Chronic pain is like… the clinical use of analogy and metaphor in the treatment of pain in children [34].

A recent commentary detailed an extensive collection of metaphors and analogies that have been used by clinicians in multiple pediatric settings (Figure 6) [34]. They focus on four explanatory categories: (1) the difference between acute and chronic pain; (2) pain transmission/spreading; (3) factors that impact pain perception; and (4) pain rehabilitation. These metaphors have been used by numerous professionals to explain pain biology, outline treatment goals, and to help patients reconceptualize the pain problem.

### 4.2. Books

Providing patients with written resources that they can utilize outside of the clinical setting can be an instrumental factor in consolidating their reconceptualization of pain. 

#### 4.2.1. Adult Pain Books

*Explain Pain and Protectometer*: The book *Explain Pain* [61] is being recognized as an invaluable resource for chronic pain patients and for professionals delivering PNE to patients. The book, written by David Butler and Lorimer Moseley, details many facets of pain biology and pain management. It is written in an approachable and engaging format, and includes illustrations throughout [61]. An education program based on the book was shown to result in lower pain scores at a three-month follow up in a group of fibromyalgia patients compared to patients who received education about activity management [62]. A pilot study also utilized the book in a session treating patients with chronic whiplash, and revealed a significant decrease in disability and an increase in pain thresholds at follow-up [63]. 

**Figure 7:**
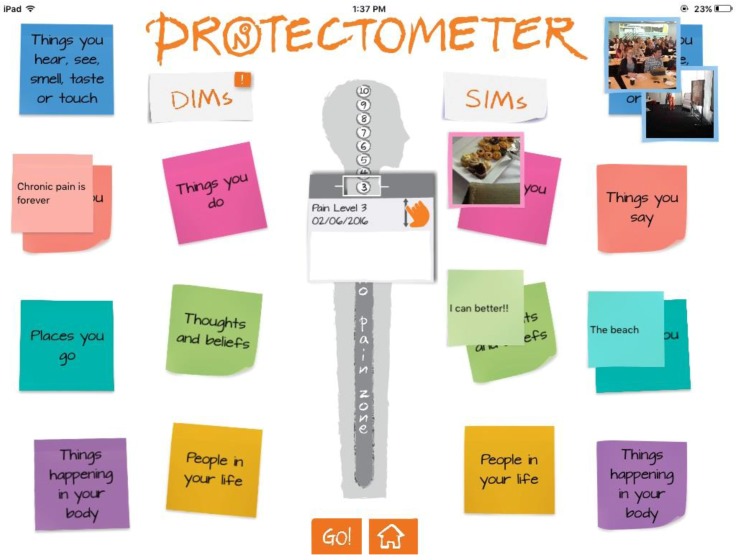
Example diagram of the ‘Protectometer’ tool provided in the available iOS app. Adapted from Protectometer: iOS Application in *Appliquette*. Available online [73].

A recently published follow-up book, *The Explain Pain Handbook: Protectometer* [64], is a patient-targeted handbook with updated information which includes an interactive pain treatment tool. The ‘Protectometer’ is a tool that allows patients to map out their pain on a day-to-day basis and identify stressors and what the authors call ‘DIMS’ (Danger(s) In Me) and ‘SIMS’ (Safety(s) In Me). There is also a ‘Protectometer’ iOS APP available (Figure 7) to build upon the activity in the handbook and to provide patients with a user-friendly way to define their ‘personal pain formula’ [64].

*Why Do I Hurt:* Another recent series of patient education books was written by physical therapist and clinical neuroscientist, Adrianne Louw. *Why Do I Hurt?* [65] is a basic patient pain neuroscience manual for chronic pain, covering pain biology and nervous system phenomena. The material is accessible for readers not already versed in the science, and includes illustrations, metaphors, and examples. Other books in his PNE series are focused on specific chronic pain problems including *Why Pelvic Pain Hurts* [66], *Your Headache Isn’t All In Your Head* [67], *Whiplash: An Alarming Message From Your Nerves* [68], and more [69,70]. There is also a workbook in the series specifically for PNE providers, *Therapeutic Neuroscience Education: Teaching Patients About Pain* [71]. This book is unique in that it is geared towards clinicians and focuses on the best ways to explain pain and demonstrate pain biology concepts. Louw has also developed the “*Why You Hurt: Therapeutic Neuroscience Education System*”, a clinical tool including colorful educational flashcards, teaching cues, pain questionnaire cards, and homework cards, all aimed at facilitating PNE [72]. The system provides an innovative way to help providers execute PNE in clinical settings. Although not written for a pediatric audience, these interactive resources hold great potential for engaging younger patients.

Despite the potential value of these materials to patients, they may not be sufficient to replace in-person PNE. In a group of adult fibromyalgia patients, written education alone did not significantly impact pain catastrophizing or functioning during daily tasks [74]. Even if patients may appreciate receiving written PNE, for it to truly be an agent of change, it may need to be delivered in person to provide an engaging, interactive format, or incorporated into a larger treatment plan that targets multiple elements of the biopsychosocial model [75].

#### 4.2.2. Pediatric Pain Books

There is no book that exclusively focuses on PNE in pediatric pain. However, there are several published books from the past decade that have been directed toward parents of youth with chronic pain, a key audience for PNE. These books comprise multiple sections that often include: PNE, education on treatment options, and an introduction to pain management skills (e.g., relaxation, behavioral activation) that parents can foster in their children. For example, “*Conquering Your Child’s Chronic Pain*” [76] introduces parents to valuable PNE topics and helpful relaxation and visualization techniques. “*Relieve Your Child’s Pain*” [77] details ways that parents can reduce stress in the home and properly evaluate their child’s pain, and addresses fears parents may have about their child’s pain problem. More recent contributions include “*Managing Your Child’s Chronic Pain*” [78], which provides parents with insight on CBT strategies as well as vignettes and stories of patient and family experiences, and “*When your child hurts*,” [34], which provides extensive PNE (over 30 pages dedicated to “Understanding What Pain is (and is Not)”) along with specific strategies geared toward breaking the cycle of the child’s pain problem. The goal of these books is to provide valuable insight and relief as parents struggle to help their child manage chronic pain. Despite the growth in these resources, there are no published studies that examine their impact on parent and child function in the context of child pain.

### 4.3. Group Education Models

When considering practical and efficient methods of delivering PNE to patients, group education is a viable option. Group models allow for a more time- and cost-efficient platform to educate patients with similar needs. The RCT by Abram and colleagues [42] described above delivered psychoeducation within a group setting, and revealed some positive effects. A group model of education may be a more efficient way to deliver information to patients instead of incorporating it into individual appointments [42]. In addition to increasing efficiency, group PNE sessions may be advantageous in that they give patients an opportunity to connect with other patients and even learn from others’ experiences. However, it has also been shown that a group model, as opposed to a one-on-one session, may suffer in efficacy [51]. The group model has the potential to prevent patients from asking questions or voicing concerns, and the one-on-one patient-clinician model may be vital for clinicians to assist in a patients’ individual reconceptualization of pain. Moreover, there are several issues to consider in relation to group composition that can potentially impact outcomes, such as size of the group, homogeneity/heterogeneity, and age (see recent review [79]). It is likely that a combination of group and one-on-one sessions may provide increased efficiency in a manner that does not sacrifice the individually tailored care that may be essential for effective pediatric pain treatment.

### 4.4. Video

**Figure 8:**
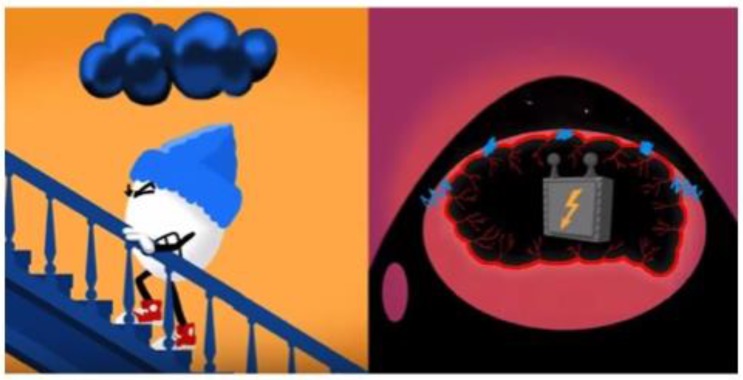
Image obtained from the online video, ‘Migraine: how it works and how to get it under control’ [80].

When presenting PNE to patients, especially in pediatrics, it is likely important to engage them using multiple modalities to facilitate processing complex and novel information. One approach is the use of short video clips. These videos are likely a familiar platform for patients to explore PNE in their own time and in comfortable home environments. One video, created by the German Pediatric Pain Center (Figure 8), explains pain for patients with migraines. Cartoons are used to explain why some people have migraines, what they mean, and how one can manage them [80]. Another video entitled “*Understanding pain in less than 5 min*” [81] uses active illustrations to present chronic pain from a biopsychosocial perspective. It is available on YouTube in over ten different languages.

## 5. Developmental Considerations and Next Steps

Research examining PNE for pediatric chronic pain is somewhat uncharted territory. Despite the clear relevance of PNE to children and young people, RCTs of PNE programs have typically been conducted with adults, and most RTCs employing psychoeduation in pediatric populations have examined psychoeducation only as part of a broader psychological treatment package and in many cases focus on pain management versus pain neurophysiology. As described above, educational resources that are appropriate for pediatric populations are currently available, such as *Explain Pain*, *The Protectometer* [64], and online animated videos. However, given that the neuroscience of pain is complex, resources that align with the child’s current state of cognitive and psychosocial development will be essential. Of particular importance for pediatrics, children who are unwell or highly stressed may also be functioning at lower cognitive levels than they otherwise would [15]. Materials must, therefore, be tailored according to both the child’s cognitive-developmental stage and his/her physical and affective state. Consideration of Piaget’s developmental stages may provide insight into the appropriate adaptation for PNE materials and delivery across early and middle childhood. The review by Jaaniste and colleagues [15] provides an excellent example of how to consider these conceptual stages when providing children with information about forthcoming medical procedures, and these examples could be usefully applied to PNE development. In order to appeal to youth at different developmental stages, resources could also be available in multiple modalities. These may be in the format of written resources, in-person dialogue models, or online animated videos and apps. Metaphors and stories should be further utilized to create specific pediatric educational tools as they provide a format for making complex concepts concrete and accessible. It is also important to consider the changing parent-child relationship across development, and recognition of the impact of parents on child learning and beliefs [82]. Most of the pain books developed for pediatric populations described above are targeted at parents, and this may indeed be an ideal method of PNE transmission for younger patients who frequently look to parents for learning opportunities.

## 6. Conclusions

PNE is receiving growing interest as an intervention in the field of chronic pain. Its potential application is vast, ranging from preoperative prevention programs to cross-disciplinary chronic pain management programs. Since PNE can be applied to multiple treatment scenarios and delivered by a variety of health professionals, its potential influence on patients is broad. Pain neuroscience education is in the midst of finding its place among a plethora of cognitive-behavioral treatments for chronic pain. A necessary next step is the inclusion of pediatric populations. Rationale for PNE, such as the detriment of irrational beliefs and maintenance of fear-avoidance, are equally relevant for younger patients. Given existing evidence for PNE in adults, this topic deserves more attention in the pediatric realm.
